# Xenobiotic-Induced Hepatocyte Proliferation Associated with Constitutive Active/Androstane Receptor (CAR) or Peroxisome Proliferator-Activated Receptor α (PPARα) Is Enhanced by Pregnane X Receptor (PXR) Activation in Mice

**DOI:** 10.1371/journal.pone.0061802

**Published:** 2013-04-23

**Authors:** Ryota Shizu, Satoshi Benoki, Yuki Numakura, Susumu Kodama, Masaaki Miyata, Yasushi Yamazoe, Kouichi Yoshinari

**Affiliations:** Division of Drug Metabolism and Molecular Toxicology, Graduate School of Pharmaceutical Sciences, Tohoku University, Aoba-ku, Sendai, Japan; Juntendo University School of Medicine, Japan

## Abstract

Xenobiotic-responsive nuclear receptors pregnane X receptor (PXR), constitutive active/androstane receptor (CAR) and peroxisome proliferator-activated receptor α (PPARα) play pivotal roles in the metabolic functions of the liver such as xenobiotics detoxification and energy metabolism. While CAR or PPARα activation induces hepatocyte proliferation and hepatocarcinogenesis in rodent models, it remains unclear whether PXR activation also shows such effects. In the present study, we have investigated the role of PXR in the xenobiotic-induced hepatocyte proliferation with or without CAR activation by 1,4-bis[2-(3,5-dichloropyridyloxy)]benzene (TCPOBOP) and phenobarbital, or PPARα activation by Wy-14643 in mice. Treatment with TCPOBOP or phenobarbital increased the percentage of Ki-67-positive nuclei as well as mRNA levels of cell proliferation-related genes in livers as expected. On the other hand, treatment with the PXR activator pregnenolone 16α-carbonitrile (PCN) alone showed no such effects. Surprisingly, PCN co-treatment significantly augmented the hepatocyte proliferation induced by CAR activation with TCPOBOP or phenobarbital in wild-type mice but not in PXR-deficient mice. Intriguingly, PXR activation also augmented the hepatocyte proliferation induced by Wy-14643 treatment. Moreover, PCN treatment increased the RNA content of hepatocytes, suggesting the induction of G0/G1 transition, and reduced mRNA levels of *Cdkn1b* and *Rbl2*, encoding suppressors of cell cycle initiation. Our present findings indicate that xenobiotic-induced hepatocyte proliferation mediated by CAR or PPARα is enhanced by PXR co-activation despite that PXR activation alone does not cause the cell proliferation in mouse livers. Thus PXR may play a novel and unique role in the hepatocyte/liver hyperplasia upon exposure to xenobiotics.

## Introduction

Xenobiotic-sensing pregnane X receptor (PXR, NR1I2) and constitutive active/androstane receptor (CAR, NR1I3) are members of the NR1I subfamily of the nuclear receptor gene superfamily. Both receptors play pivotal roles in the xenobiotic-induced expression of genes encoding drug-metabolizing enzymes and transporters, such as CYP3As and CYP2Bs, UDP-glucoronosyltransferases, sulfotransferases, glutathione-*S*-transferases, and ATP-binding cassette transporters [1], [2], [3]. These receptors exhibit some degree of overlapping properties: They form heterodimers with the retinoid X receptor (RXR, NR2B1) and bind to common regulatory sequences in the regulatory regions of their target genes, thereby regulating distinct but overlapping sets of genes. Thus, PXR and CAR work in concert to protect the body against harmful xenobiotics.

Recent studies have expanded biological and pathophysiological functions of PXR and CAR. They are known to regulate hepatic energy metabolism by cross-talking with regulators of energy homeostasis [4], [5]. In addition, CAR has been reported to promote hepatocarcinogenesis in response to xenobiotics in mice through inducing cell proliferation and suppressing apoptosis without DNA lesions (see below). In contrast, it remains unclear whether PXR has such functions despite the functional similarities with CAR.

Phenobarbital (PB), a well-known activator of CAR, is also well established as a liver tumor promoter in rodents, causing liver tumors in experimental rodent models via a nongenotoxic mode of action [6], [7]. Yamamoto et al. have successfully demonstrated using CAR-deficient mice that CAR is an essential factor for PB-induced liver tumor formation following the initiation with diethylnitrosamine [8]. To date, many groups have reported possible mechanisms for the CAR-mediated hepatocyte proliferation in mice. For example, CAR induced the transcription of the genes encoding modulators of p53 tumor suppressor protein, such as *Gadd45* and *Mdm2* [9], [10], [11]. Another report demonstrated that CAR-induced hepatocyte hyperplasia was mediated by the expression of the oncogene *c-Myc* and its target *Foxm1* [12]. However, the entire machinery of the hepatocellular carcinoma formation promoted by CAR in rodents has not been elucidated. Moreover, its relevance to human health is still controversial due to the lack of clear information on the molecular mechanism.

PXR activators have long been known to increase liver weight without observable increase in cell proliferation [13] while a recent report demonstrated that intraperitoneal administration of pregnenolone 16α-carbonitrile (PCN), an activator of rodent PXR, at a high dose (400 mg/kg, 4 days) increased the number of proliferating cell nuclear antigen (PCNA)-positive nuclei in mouse livers [14]. Since PCNA expression increases in G1/S phases [15] and the authors of the report have not investigated other cell proliferation-related markers, it remains unclear whether PXR activation is able to induce hepatocyte proliferation as is CAR or not. In fact, it has been reported that PXR up-regulates the protein levels of cyclin-dependent kinase (CDK) inhibitor p21 to suppress the proliferation of colon cancer cells [16] and that ectopic PXR expression in neuroblastoma cells resulted in growth suppression [17]. In our preliminary experiments, using a quantitative reverse transcription-PCR (RT-PCR) analysis, we found that hepatic mRNA levels of some cell cycle-associated genes including *Foxm1* and *Ccnd1* (Cyclin D1) were increased in mice by treatment with the murine CAR ligand 1,4-bis[(3,5-dichloropyridin-2-yl)oxy]benzene (TCPOBOP) but not with PCN (Yoshinari et al. unpublished results). Since PXR is activated by a wide range of xenobiotics far more than CAR [1], [3], the elucidation of the PXR’s ability to initiate hepatocyte proliferation is quite important for the chemical safety evaluation.

In addition to the CAR activators, ligands for peroxisome proliferator-activated receptor α (PPARα, NR1C1), another member of the nuclear receptor superfamily, have been identified as nongenotoxic carcinogens in rodents [18], [19], [20]. In the present study, we have investigated the influence of PXR activation on hepatocyte proliferation and the role of PXR in the xenobiotic-induced hepatocyte proliferation mediated by CAR or PPARα in mice.

## Materials and Methods

### Ethics Statement

The animal experiments were approved by the Institutional Animal Care and Use Committee at Tohoku University (Sendai, Japan). All experiments were performed in accordance with the Guidelines for Animal Experiments of Tohoku University (Sendai, Japan).

### Materials

TCPOBOP, PCN, Wy-14643, propidium iodide (PI), Pyronin Y, 7-aminoactinomycin D (7-AAD) and collagenase (type IV) were obtained from Sigma-Aldrich (St. Louis, MO). PB sodium salt and corn oil were purchased from Wako Pure Chemical Industries (Osaka, Japan). Saline for injection was purchased from Otsuka Pharmaceuticals (Tokyo, Japan). RNase A was purchased from Nacalai Tesque (Kyoto, Japan). Oligonucleotides were commercially synthesized by Fasmac (Atsugi, Japan). All other chemicals were of the highest grade available from Wako Pure Chemical Industries or Sigma-Aldrich.

### Animal Treatment

Male wild-type (C57BL/6, Charles River Japan, Yokohama, Japan) and *Pxr*-null mice (gift from Dr. Staudinger, University of Kansas, Lawrence, KS) [21] were maintained in a temperature- and light-controlled environment (24°C, 12 h-light and 12 h-dark cycle). Mice (around 8 weeks old) were intraperitoneally treated with vehicle (corn oil) or PCN (100 mg/kg) in combination with or without TCPOBOP (3 mg/kg), PB (100 mg/kg) or Wy-14643 (150 mg/kg), or fed a diet (CE-2, Clea Japan, Tokyo, Japan) containing 1000 ppm PB, 500 ppm PCN or both for 1 week. Then, mice were sacrificed by cervical dislocation, from which livers were excised and weighed.

### Determination of mRNA Levels

Total RNA was individually isolated from livers using the acid guanidine–phenol–chloroform method. mRNA levels were determined by real-time RT-PCR analysis and PCR-array analysis. For real-time RT-PCR analysis, first-stranded cDNA was individually synthesized with High Capacity cDNA Reverse Transcription Kit (Applied Biosystems, Foster City, CA). Quantitative RT-PCR was performed using the Power SYBR Green PCR Master Mix (Applied Biosystems) and primer pairs for genes of interest (Table S1). The mRNA levels were normalized with those for *Actb* (β-actin) and the relative mRNA levels in control groups were set at 1. For PCR-array analysis, hepatic total RNA prepared from individual mice was pooled for cDNA synthesis using RT^2^ First-strand Kit (Qiagen, Valencia, CA). Comprehensive analysis of mRNA levels of cell cycle-associated genes was performed using the Mouse Cell Cycle RT^2^ Prolifer PCR Array (Qiagen) according to the manufacture’s protocol.

### Histology and Immunohistochemistry

Livers were fixed in 10% neutral buffered formalin (Wako Pure Chemicals). Sections were stained with anti-Ki-67 antibody and counter stained with hematoxilin using standard procedures by Morpho Technology (Sapporo, Japan). Image capture and acquisition were carried out with a Leica DMLB microscope and Leica DC viewer software (Leica Microsystems Wetzlar GmbH, Wetzlar, Germany). Image J software (U. S. National Institutes of Health, Bethesda, MA) was used for the analysis of data. The proliferation index was established as follows: total and Ki-67-positive nuclei were counted in randomly selected five areas (magnification; ×100) per each section from individual mouse and calculated the percentage of Ki-67-positive nuclei for each mouse. Then, the mean and SD values for each experimental group was calculated.

### Flow Cytometry for Cell Cycle Analysis

C57BL/6 mice (around 8 weeks old) were intraperitoneally treated with vehicle (corn oil), PCN (100 mg/kg) or TCPOBOP (3 mg/kg), and 48 h later hepatocytes were isolated from the livers by a two-step collagenase perfusion method according to the Seglen’s report [22]. Parenchymal hepatocytes were isolated by centrifuging the single cell suspension at 50×g for 5 min [23]. Cell cycle analysis was performed by staining DNA with PI or double-staining with 7-AAD and Pyronin Y for DNA and RNA, respectively. Briefly, mouse primary hepatocytes were fixed in 70% ethanol for 30 min on ice. For PI staining, cells were incubated with 200 µg/mL RNase A for 30 min at 37°C and then with 20 µg/mL PI for overnight on ice. For DNA/RNA double-staining, cells were incubated with 25 µg/mL 7-AAD for 30 min at room temperature and then with 4 µg/mL Pyronin Y for 10 min on ice. These cells were washed with phosphate-buffered saline and then analyzed on a FACSCalibur flow cytometer (BD Biosciences, San Jose, CA). Data were analysed with CellQuest software (BD Biosciences).

### Statistical Analysis

Statistical analysis was performed using GraphPad Prism (GraphPad Software, La Jolla, CA). All data are provided as the mean ± SD. The one-way analysis of variance followed by Tukey-Kramer test was performed to compare multiple experimental groups. Values of *P*<0.05 were considered statistically significant.

## Results

### Influence of PXR Activation on the Hepatocyte Proliferation with or without CAR Activation

**Figure 1:**
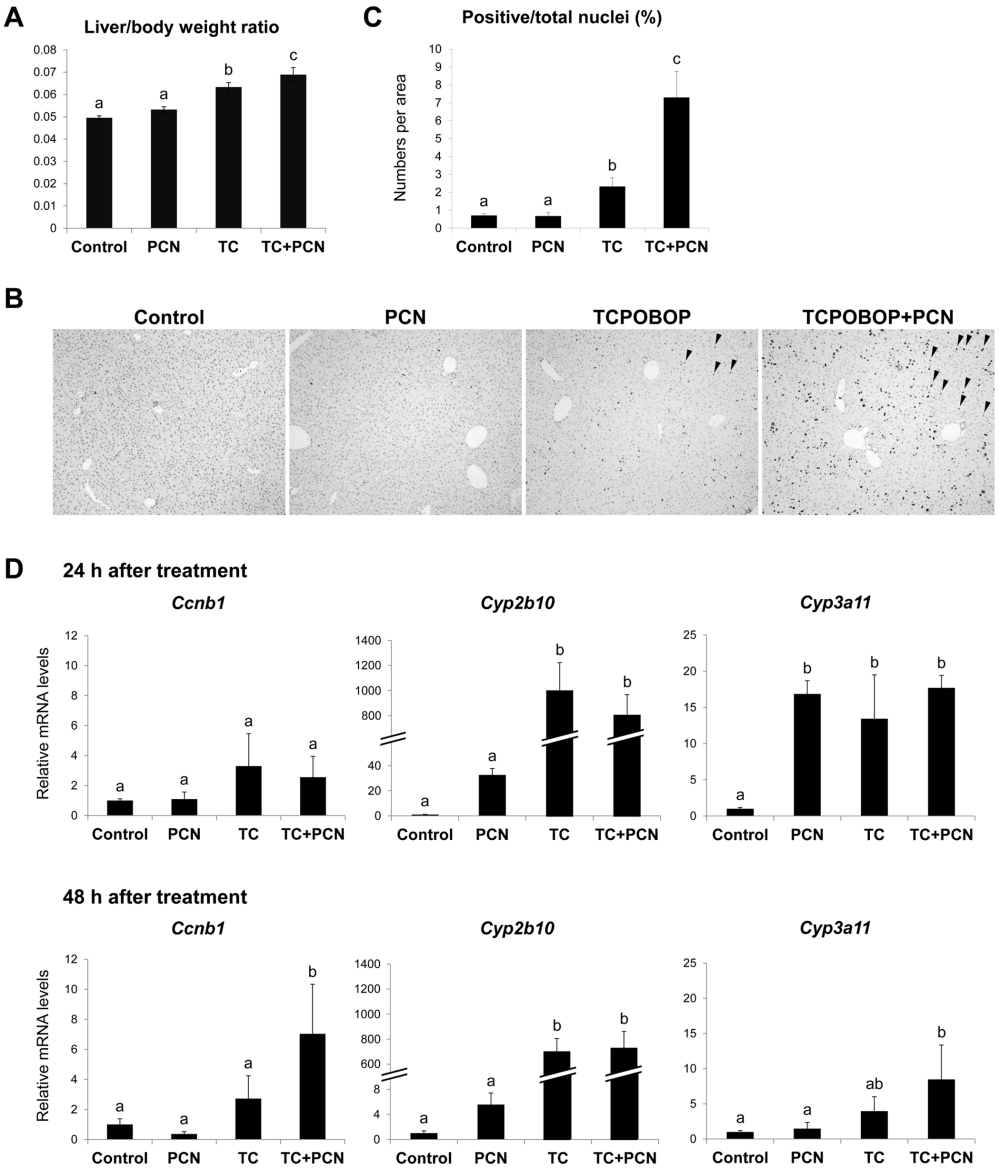
Hepatocyte proliferation after PCN and/or TCPOBOP treatment in mice. Male mice were treated intraperitoneally with vehicle (corn oil; Control), TCPOBOP (TC; 3 mg/kg), PCN (100 mg/kg) or both for 48 h. (A) The liver to body weight ratios were calculated. (B) Livers were fixed and stained with anti-Ki-67 antibody for the proliferating cell nuclei. Arrowheads indicate Ki-67-positive nucleus. (C) The percentage of Ki-67-positive nuclei was calculated as described in Materials and Methods. (D) Total hepatic RNAs were subjected to quantitative RT-PCR for *Cyp2b10, Cyp3a11* and *Ccnb1*. Values are the mean ± SD (n = 3 or 4). Columns not sharing a common letter (a, b and c) differ significantly with each other (*P*<0.05; Tukey-Kramer test).

To investigate the influence of PXR activation on hepatocyte proliferation, mice were treated intraperitoneally with PCN (100 mg/kg) in combination with or without TCPOBOP (3 mg/kg). TCPOBOP but not PCN treatment increased the liver to body weight ratio by 28% 48 h after treatment (Fig. 1A). Co-treatment with PCN and TCPOBOP further increased the ratio to 139% that of control (Fig. 1A). Immunohistopathological analyses of the liver were carried out using antibody against Ki-67 and hematoxylin (Fig. 1B, C). We used Ki-67 rather than PCNA as a marker for the hepatocyte proliferation in this study because PCNA and Ki-67 levels become maximal at G1/S and G2/M phases, respectively, and Ki-67 is more tightly associated with mitosis than PCNA [15], [24], [25]. The percentage of Ki-67-positive nuclei was significantly increased 48 h after TCPOBOP treatment. In contrast, PCN treatment did not affect it. However, PCN co-treatment with TCPOBOP more significantly increased the percentage of Ki-67 positive nuclei than did TCPOBOP treatment alone. Real-time RT-PCR analysis confirmed the similar changes in hepatic mRNA levels of *Ccnb1*, encoding Cyclin B1. TCPOBOP but not PCN treatment increased them and co-treatment with PCN and TCPOBOP further increased them (Fig. 1D). In contrast to the markers for cell proliferation, PCN co-treatment did not enhance the TCPOBOP-mediated increase in the mRNA levels of *Cyp2b10*, a representative target gene of CAR, 24 and 48 h after treatment (Fig. 1D). Treatment of mice with PCN increased the mRNA levels of *Cyp3a11*, a representative target gene of PXR, at 24 h, indicating that the treatment did activate PXR (Fig. 1D).

To further confirm the influences of the chemical treatment on the hepatocyte proliferation, we determined mRNA levels of a variety of genes associated with cell cycle using PCR-array system, and found that PCN treatment did not increase hepatic mRNA levels of cell cycle-associated genes such as *Ccna2, Ccnb1, Mcm2 or Mki67*, which were increased with TCPOBOP treatment (Table S2). Again, PCN co-treatment further increased these levels (Table S2).

While TCPOBOP directly binds and activates mouse CAR, PB indirectly activates CAR through an unidentified cellular signaling pathway [26]. We thus investigated whether PCN treatment could also augment the hepatocyte proliferation induced by PB treatment (Fig. S1). Single PB treatment marginally increased the liver to body weight ratio and the percentage of Ki-67-positive nuclei, but PCN co-treatment drastically increased these levels. These results clearly suggest that upon activation with PCN, PXR enhances the CAR-mediated hepatocyte proliferation independent of the type of CAR activators.

**Figure 2:**
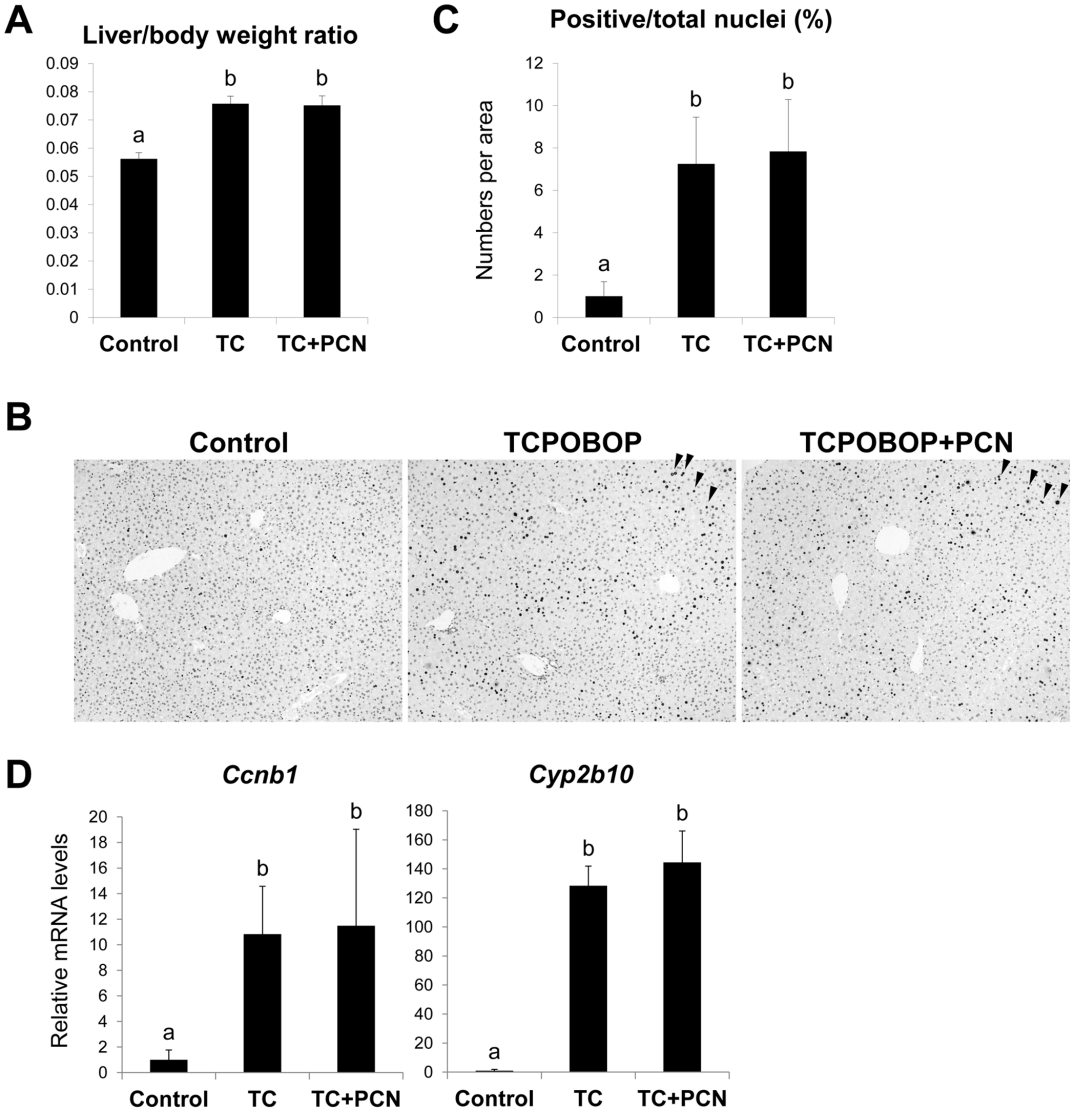
Influences of PCN co-treatment on the hepatocyte proliferation induced by TCPOBOP treatment in PXR-deficient mice. Male *Pxr^−/−^* mice were treated intraperitoneally with vehicle (corn oil; Control), TCPOBOP (TC; 3 mg/kg), PCN (100 mg/kg) or both for 48 h. (A) The liver to body weight ratios were calculated. (B) Livers were fixed and stained with anti-Ki-67 antibody. Arrowheads indicate Ki-67-positive nucleus. (C) The percentage of Ki-67-positive nuclei was calculated as described in Materials and Methods. (D) Total RNAs extracted from the liver were subjected to quantitative RT-PCR for *Cyp2b10* and *Ccnb1*. Values are the mean ± SD (n = 4). Columns not sharing a common letter (a and b) differ significantly with each other (*P*<0.05; Tukey-Kramer test).

To investigate whether PXR is the factor that mediates the enhancing effects, we next performed similar experiments using PXR-deficient mice with TCPOBOP and PCN. As observed in wild-type mice, liver to body weight ratios were increased 48 h after TCPOBOP administration in *Pxr*-null mice (135% that of control) (Fig. 2A). However, co-treatment with PCN had no effect on these TCPOBOP-induced changes (Fig. 2A). Moreover, neither the percentage of Ki-67-positive nuclei nor *Ccnb1* mRNA levels was enhanced by the co-treatment with PCN in the livers of TCPOBOP-treated *Pxr*-null mice (Fig. 2B–D). CAR activation following TCPOBOP treatment in these mice was confirmed by increases in *Cyp2b10* and *Cyp3a11* mRNA levels (Fig. 2D and data not shown).

**Figure 3:**
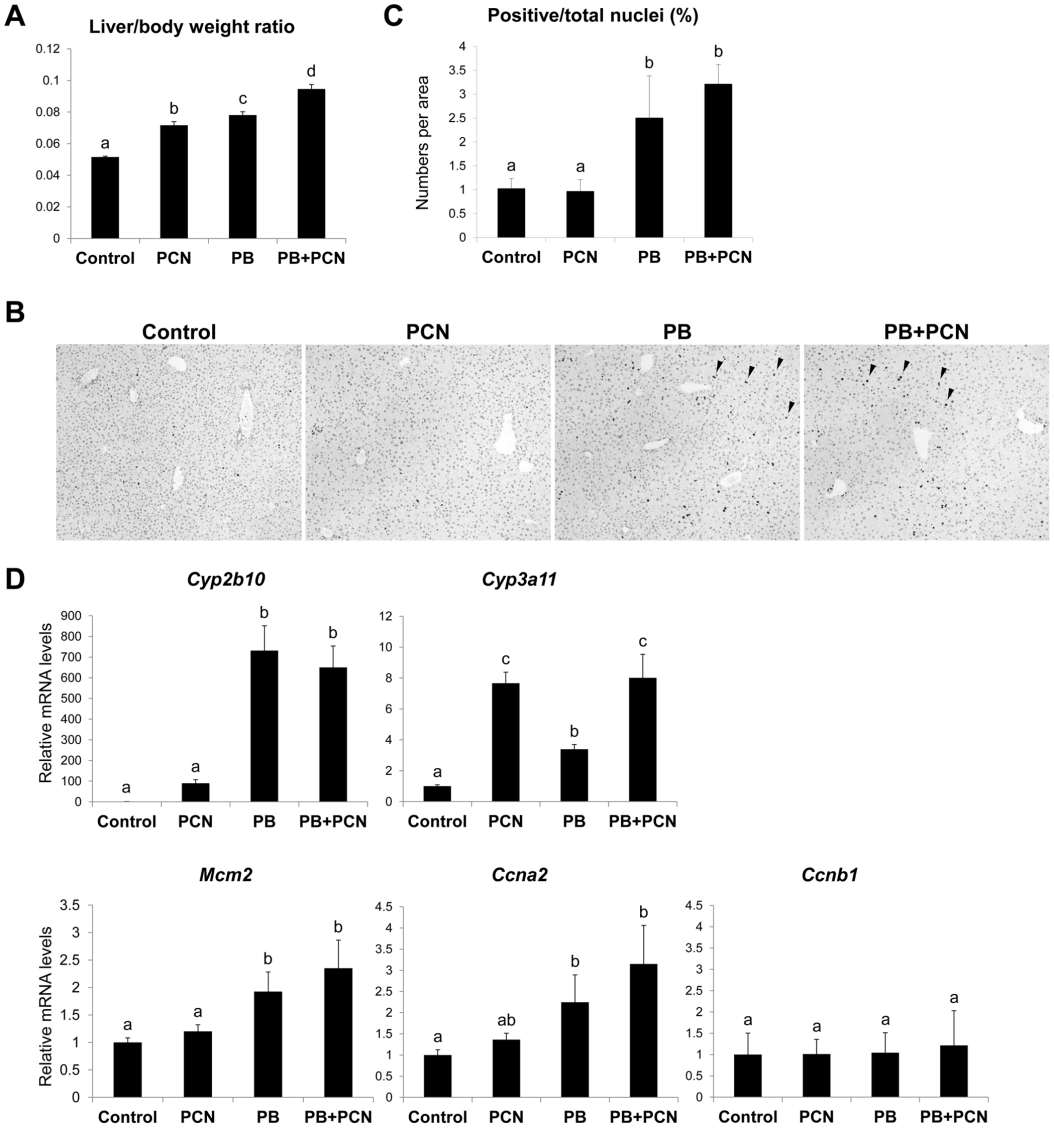
Influences of 1-week feeding with PCN and/or PB on the hepatocyte proliferation. Male mice were fed a normal diet (Control) or a diet containing PB (1000 ppm), PCN (500 ppm) or both for 1 week. (A) The liver to body weight ratios were calculated. (B) Livers were fixed and stained with anti-Ki-67 antibody. Arrowheads indicate Ki-67-positive nucleus. (C) The percentage of Ki-67-positive nuclei was calculated as described in Materials and Methods. (D) Total RNAs extracted from the liver were subjected to quantitative RT-PCR for *Cyp2b10, Cyp3a11, Mcm2, Ccna2* and *Ccnb1*. Values are the mean ± SD (n = 4). Columns not sharing a common letter (a, b, c and d) differ significantly with each other (*P*<0.05; Tukey-Kramer test).

To confirm that PXR activation alone does not initiate hepatocyte proliferation in mouse livers, we investigated the influence of continuous PXR activation on the hepatocyte proliferation, feeding mice with a normal diet or a diet containing PCN (500 ppm) and/or PB (1000 ppm) for a week. Under these conditions, hepatic mRNA levels of *Cyp2b10* and *Cyp3a11* were significantly increased by each chemical treatment (Fig. 3D). The liver to body weight ratios were increased by either PCN or PB administration (by 39% and 58%, respectively) and further increased by co-treatment (184% that of control) (Fig. 3A). The percentage of Ki-67-positive nuclei was increased by PB treatment, and PCN co-treatment tended to enhance it although the data did not meet statistical significance (Fig. 3B, C). Importantly, 1-week treatment with PCN did not increase the percentage of Ki-67-positive nuclei (Fig. 3B, C). Hepatic *Ccnb1* mRNA levels were unchanged with either treatment (Fig. 3D). mRNA levels of *Mcm2* encoding Minichromosome maintenance protein 2 or MCM2, which is up-regulated in S-phase of cell cycle and acts to initiate DNA synthesis, and *Ccna2* encoding Cyclin A2 were increased with PB or PB/PCN treatment but not PCN alone (Fig. 3D).

### Influence of PCN Treatment on the PPARα-dependent Hepatocyte Proliferation

**Figure 4:**
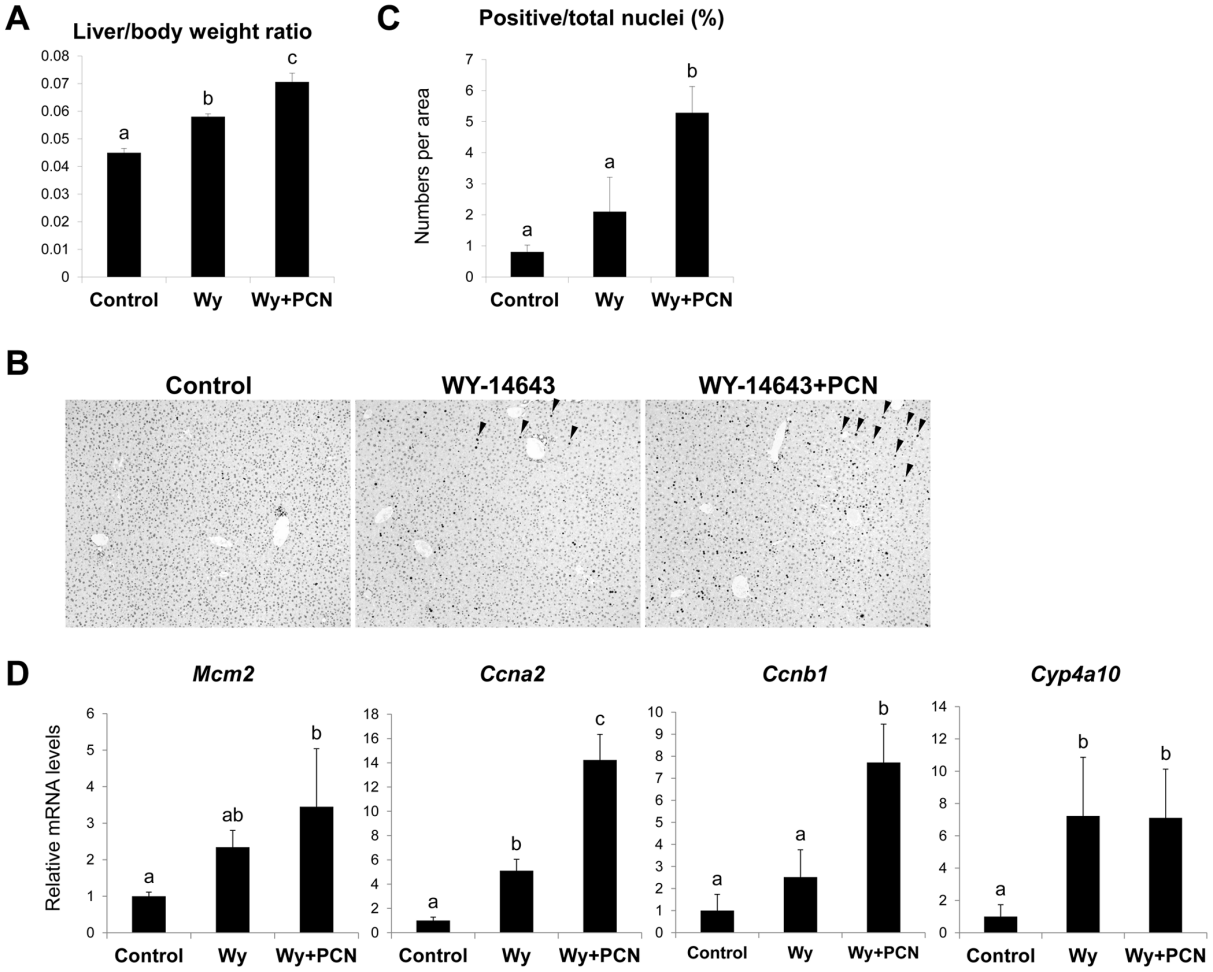
Influences of PCN co-treatment on the hepatocyte proliferation induced by single Wy-14643 treatment. Male mice were treated intraperitoneally with vehicle (corn oil; Control) or Wy-14643 (Wy; 150 mg/kg) in combination with or without PCN (100 mg/kg) for 48 h. (A) The liver to body weight ratios were calculated. (B) Livers were fixed and stained with anti-Ki-67 antibody. Arrowheads indicate Ki-67-positive nucleus. (C) The percentage of Ki-67-positive nuclei was calculated as described in Materials and Methods. (D) *Cyp4a10, Mcm2, Ccna2* and *Ccnb1* mRNA levels were determined by quantitative RT-PCR. Values are the mean ± SD (n = 3 or 4). Columns not sharing a common letter (a, b and c) differ significantly with each other (*P*<0.05; Tukey-Kramer test).

We next investigated whether PXR activation by PCN could enhance the hepatocyte proliferation induced by other signals. For this purpose, we have focused on PPARα, which has been reported to induce hepatocyte proliferation in rodents by activating signal(s) other than ones activated by CAR [27], [28]. Intraperitoneal treatment of mice with the PPARα ligand Wy-14643 for 48 h increased the liver to body weight ratios (by 29%) and co-treatment with PCN further increased it (157% that of control) (Fig. 4A). Wy-14643 treatment alone tended to increase the percentage of Ki-67-positive nuclei as well as hepatic mRNA levels of *Mcm2*, *Ccna2* and *Ccnb1*, indicating that the treatment induced hepatocyte proliferation as expected (Fig. 4B–D). Intriguingly, PCN co-treatment further increased these levels (Fig. 4B–D). In contrast, Wy-14643 treatment increased mRNA levels of *Cyp4a10*, a representative PPARα target gene, but PCN co-treatment did not further increase them (Fig. 4D).

### Influence of PCN Treatment on the G0/G1 Transition of Hepatocytes

**Figure 5:**
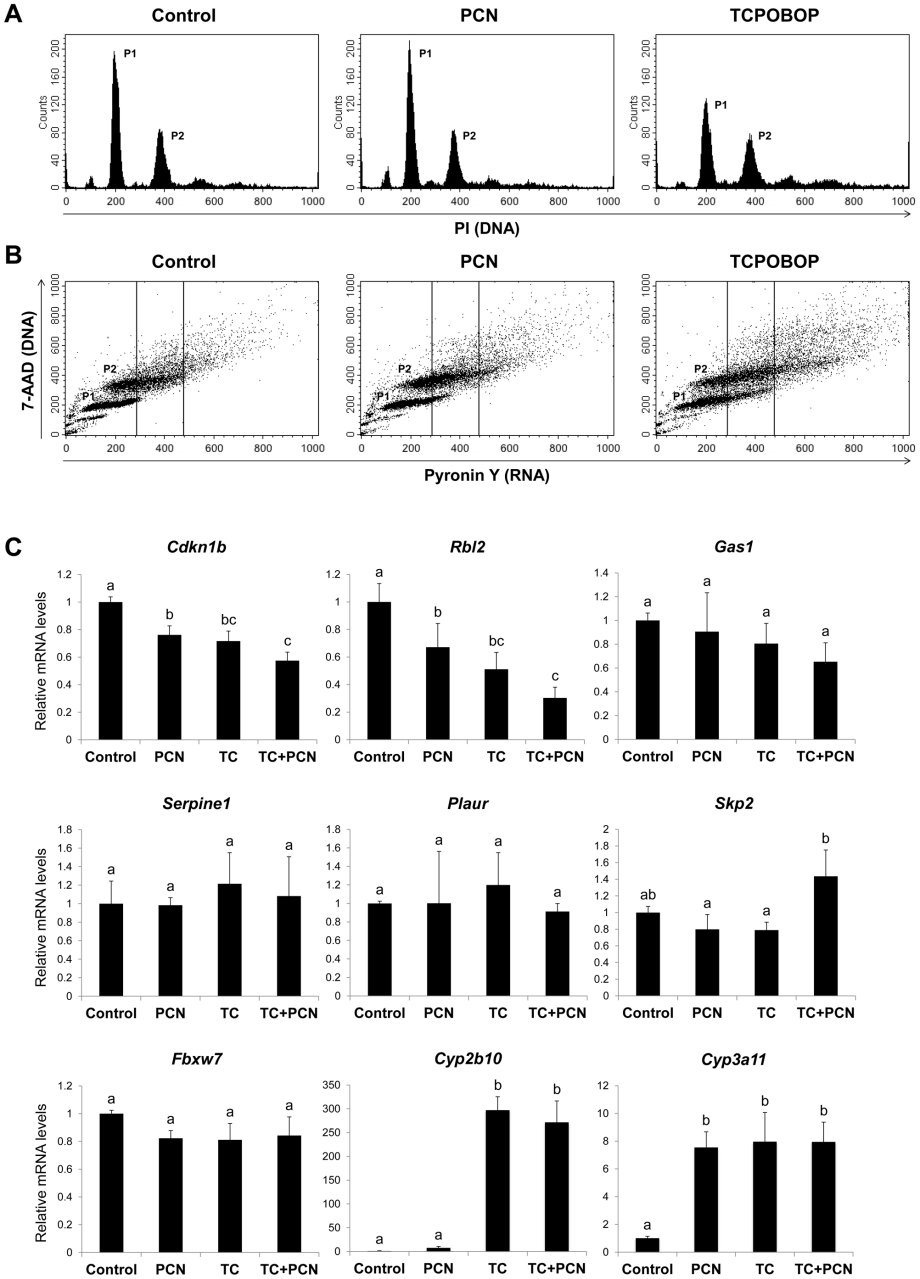
Influence of PCN treatment on the G0/G1 transition of mouse hepatocytes. (A, B) Male mice were treated intraperitoneally with vehicle (corn oil; Control), PCN (100 mg/kg) or TCPOBOP (3 mg/kg) and primary hepatocytes were isolated by collagenase perfusion method from the liver 48 h after treatment. Fixed cells were incubated with PI for DNA staining to determine cell cycle distribution (A) or with 7-AAD and Pyronin Y for DNA and RNA staining, respectively, to separate G0 and G1 phase cells (B) by flow cytometry. P1 and P2 fractions represent G0/G1-phase 4 n hepatocytes and G2/M-phase 4 n or G0/G1-phase 8 n hepatocytes, respectively. One set of representative results among 4 independent experiments is shown. (C) Male mice were treated intraperitoneally with vehicle (corn oil; Control), TCPOBOP (TC; 3 mg/kg) and/or PCN (100 mg/kg) for 24 h. Total RNAs extracted from the liver were subjected to quantitative RT-PCR for the indicated genes. Columns not sharing a common letter (a, b and c) differ significantly with each other (*P*<0.05; Tukey-Kramer test).

In this study, PXR activation did not induce the hepatocyte proliferation in mice by itself whereas it enhanced the cell proliferation induced by CAR or PPARα. We thus hypothesized that PXR activation leads quiescent (G0 phase of cell cycle) hepatocytes to enter G1 phase, making hepatocytes more sensitive to CAR or PPARα activators for cell cycle progression. To test this possibility, we investigated the influence of PCN treatment on the G0/G1 transition of mouse hepatocytes using a flow cytometer after staining DNA and RNA. In this method, G0- and G1-phase cells can be separated based on DNA and RNA contents, because quiescent G0-phase cells have a low RNA content and RNA is accumulated as cells move from G0 to G1 phase [29]. When cell cycle distribution was analyzed by DNA staining with PI, TCPOBOP treatment decreased the number of cells in P1 fraction (considered as 4 n hepatocytes in G0/G1 phases) and increased those in P2 fraction (considered as 4 n hepatocytes in G2/M phases or 8 n hepatocytes in G0/G1 phases), but PCN treatment caused no obvious changes (Fig. 5A). However, PCN treatment as well as TCPOBOP treatment increased the RNA content of both P1 and P2 hepatocytes as indicated by a shift to the right side (Fig. 5B), suggesting that a portion of the cells in these fractions entered G1 phase.

We then investigated influences of PXR activation on the expression levels of genes associated with the G0–G1 transition, namely *Cdkn1b, Rbl2, Gas1, Serpine1, Plaur, Skp2* and *Fbxw7* (Fig. 5C). PCN treatment as well as TCPOBOP treatment of mice for 24 h decreased hepatic mRNA levels of *Cdkn1b* and *Rbl2*, encoding p27 and p130, respectively, but did not affect those of other genes. As expected, mRNA levels of *Cyp2b10* and *Cyp3a11* were significantly increased by each chemical treatment.

## Discussion

CAR is known as a key transcription factor in the xenobiotic-induced hepatocyte proliferation while it remains unclear whether PXR has such a function. In this study, no proliferation was observed after PXR activation via either intraperitoneal treatment with PCN (100 mg/kg) for 48 h or feeding a diet containing PCN (500 ppm) for 1 week, suggesting that PXR activation itself does not induce the hepatocyte proliferation in mice. However, PCN treatment augmented the CAR-mediated hepatocyte proliferation induced by either TCPOBOP or PB treatment. Moreover, these enhancing effects of PCN co-treatment were not observed in PXR-deficient mice. It is therefore suggested that PXR, when activated, has a very unique function in the cell cycle of murine hepatocytes, enhancing the CAR-mediated hepatocyte proliferation without inducing the proliferation by itself.

Continuous administration of PB increased liver weight and DNA synthesis in mouse livers at day 7 as well as day 3 [30]. Consistently, after 1-week treatment with PB, the percentage of Ki-67-positive nuclei was increased in this study. However, there was no statistically significant difference in the percentage of Ki-67-positive nuclei between mice administrated both PB and PCN and mice treated with PB alone. Taken together with the results of the single dose experiments, it is suggesed that PXR activation does not continuously enhances the CAR-mediated hepatocyte proliferation and rather it enhances the early stage of the proliferation.

In addition to CAR, PPARα activation also induces hepatocyte proliferation in rodents, and the PPARα-mediated proliferation is considered to be regulated through a signal different from that for the CAR-mediated proliferation [27], [28]. Interestingly, PCN co-treatment also augmented the hepatocyte proliferation induced by the treatment with the PPARα ligand Wy-14643 in this study. Meanwhile, PCN co-treatment did not potentiates the increase in the expression of CAR or PPARα target genes (namely *Cyp2b10* and *Cyp4a10*, respectively). These results suggest that PXR does not simply enhance the CAR- or PPARα-mediated gene transcription (or repression) in hepatocytes.

At this moment, it is unclear how PXR co-activation enhances the CAR- or PPARα-related cell proliferation in mouse livers. In this study, PXR activation alone showed no effects on the expression of cell proliferation-related genes investigated such as *Ccnb1*, suggesting that PXR does not induce hepatocyte division in contrast to CAR and PPARα. Moreover, we have demonstrated that PCN treatment increased the RNA content of quiescent cells and decreased hepatic mRNA levels of *Cdkn1b* (p27) and *Rbl2* (p130), both of which negatively regulate the cell cycle transition from G0 to G1 phase [31], [32]. Loss of functional p130 promoted the development of small-cell lung carcinoma in RB- and p53-mutated mice [33]. On the other hand, overexpression of p130 in HepG2 cells led to the growth suppression, cell cycle arrest in G0/G1, and reduction in tumorigenicity in SCID mice [34]. In p27-deficient mouse, long-term treatment with PB following the initiation with diethylnitrosamine significantly promoted liver tumorigenesis compared to wild-type mice [35]. Taken together, we have hypothesized that PXR activation can let hepatocytes enter G1 phase from G0 phase through down-regulating p27 and p130 expression and make hepatocytes to divide easily. We are currently working on this hypotesis to clarify whether the PXR-mediated intracellular signaling(s) is associated with the G0/G1 transition.

Recently, Staudinger et al. reported that intraperitoneal administration to mice of PCN at a higher dose (400 mg/kg) for 4 days increased hepatic levels of PCNA [14]. Since PCNA is involved in replicative DNA synthesis and highly expressed during G1-S phases [15], their results suggest that activated PXR can move hepatocytes from G0 phase to G1/S phases. In addition, Ouyang et al. have suggested that PXR activation induces p21 protein expression and suppresses the proliferation of colon cancer cells [16]. p21 is a tumor suppressor protein which induces G1/S arrest [36], and p21-mediated inhibition of cell cycle progression is obtained by not only CDK inhibition but also direct binding to PCNA [36], [37], thereby interfering with PCNA-dependent DNA synthesis and keeping hepatocytes at G1 phase. These facts have raised a possibility that PXR activation induces not only G0/G1 transition of hepatocytes but G1/S arrest as well. Although it needs to be clarified, this hypothesis is consistent with our present findings and the findings by Staudinger et al.

In summary, we have demonstrated a new and unique role of PXR in the hepatocyte proliferation in mice. In contrast to CAR and PPARα, PXR activation alone had no obvious effects on the hepatocyte proliferation in mice. However, the co-activation of PXR significantly enhanced the CAR- or PPARα-mediated proliferation of murine hepatocytes. Since CAR or PPARα activators such as PB and fibrates are known as liver tumor promoters in rodents, our present findings suggest that PXR activators act as “enhancers” or “accelerators” in chemical carcinogenesis through enhancing the promoting abilities of CAR and PPARα although this possibility remains to be investigated in animal carcinogenesis studies in future. Our findings will thus bring a new insight into not only the molecular mechanism for the xenobiotic-mediated hepatocyte proliferation but chemical safety evaluation as well.

## Supporting Information

Figure S1 **Influence of PCN co-treatment on the hepatocyte proliferation induced by single PB treatment.** Male mice were treated intraperitoneally with vehicle (corn oil and saline; Control), PB (in saline, 100 mg/kg), PCN (in corn oil, 100 mg/kg) or both for 48 h. (A) The liver to body weight ratios were calculated. (B) Livers were fixed and stained with anti-Ki-67 antibody for the proliferating cell nuclei. (C) The percentage of Ki-67-positive nuclei was calculated as described in Materals and Methods. Values are the mean ± SD (n = 4). Columns not sharing a common letter (a, b and c) differ significantly with each other (*P*<0.05; Tukey-Kramer test).
(TIF)

Table S1 **Primers used for quantitative RT-PCR are shown.**
(DOC)

Table S2 **Changes in the gene expression levels after PCN and/or TCPOBOP treatment in mouse livers.**
(DOC)
