# Supplementary figures and images for: Xenobiotic-Induced Hepatocyte Proliferation Associated with Constitutive Active/Androstane Receptor (CAR) or Peroxisome Proliferator-Activated Receptor α (PPARα) Is Enhanced by Pregnane X Receptor (PXR) Activation in Mice
Source: PLoS One. 2013 Apr 23;8(4):e61802. doi: 10.1371/journal.pone.0061802 (PMC3634023; doi:10.1371/journal.pone.0061802)

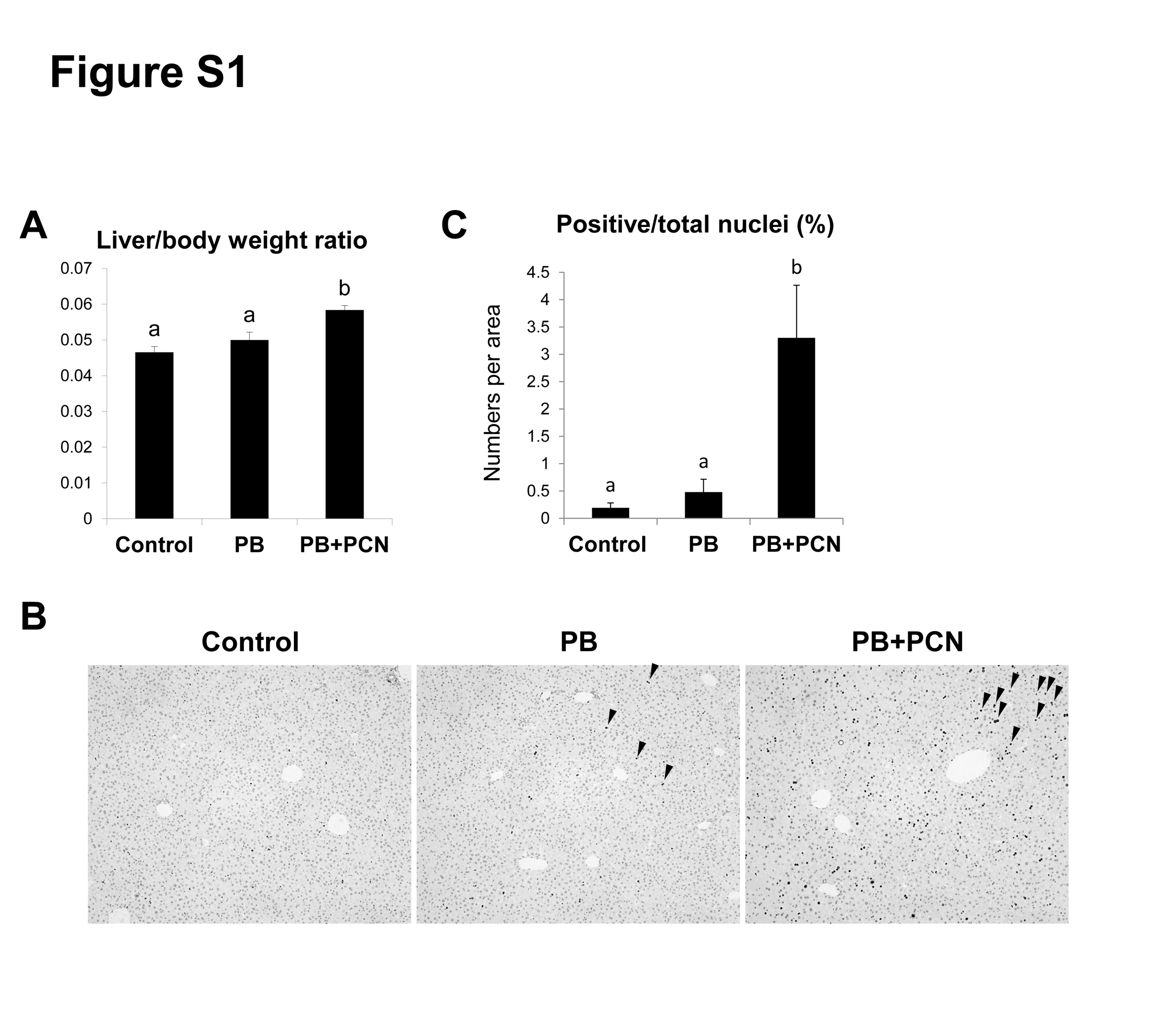

Supplement: Figure S1 — Influence of PCN co-treatment on the hepatocyte proliferation induced by single PB treatment. Male mice were treated intraperitoneally with vehicle (corn oil and saline; Control), PB (in saline, 100 mg/kg), PCN (in corn oil, 100 mg/kg) or both for 48 h. (A) The liver to body weight ratios were calculated. (B) Livers were fixed and stained with anti-Ki-67 antibody for the proliferating cell nuclei. (C) The percentage of Ki-67-positive nuclei was calculated as described in Materals and Methods. Values are the mean ± SD (n = 4). Columns not sharing a common letter (a, b and c) differ significantly with each other (P<0.05; Tukey-Kramer test). (TIF) [file pone.0061802.s001.tif]
