# Supplementary material for: Xenobiotic-Induced Hepatocyte Proliferation Associated with Constitutive Active/Androstane Receptor (CAR) or Peroxisome Proliferator-Activated Receptor α (PPARα) Is Enhanced by Pregnane X Receptor (PXR) Activation in Mice
Source: PLoS One. 2013 Apr 23;8(4):e61802. doi: 10.1371/journal.pone.0061802 (PMC3634023; doi:10.1371/journal.pone.0061802)
Supplement: Table S1 — Primers used for quantitative RT-PCR are shown. (DOC) [file pone.0061802.s002.doc]

**Table S1.** **Primers used for quantitative RT-PCR are shown.**

| Gene | Forward primer (5’ to 3’) | Reverse primer (5’ to 3’) |
| --- | --- | --- |
| *Actb* | GCCAACACAGTGCTGTTCTG | CCTGCTTGCTGATCCACATC |
| *Ccna2* | TACCCCCCAGAAGTAGCAG | GGGTCAGCATCTATCAAAC |
| *Ccnb1* | TTGACAACGGTGAATGGACAC | TGCACGGCCTTAGACAAATTC |
| *Cdkn1b* | GGGTCTCAGGCAAACTCTGAG | TGTTTACGTCTGGCGTCGAAG |
| *Cyp2b10* | AAAGTCCCGTGGCAACTTCC | CATCCCAAAGTCTCTCATGG |
| *Cyp3a11* | ACAAGCAGGGATGGACCTGG | TGTGACAGCAAGGAGAGGCG |
| *Cyp4a10* | TGTCCCAGGCATTGTCAGAGA | CCTTCGGGTTGTGGTGGAGA |
| *Fbxw7* | GCAGCCAATGGGCAAGGGCA | TGGTCCGCTCCAGCTCTGAAACA |
| *Gas1* | GCGGGATGCCAGAGCTGCGA | AGGCGCTCAGTGCCGTTCCG |
| *Mcm2* | TCACGGTGCGCCACATCGAG | CCGGGCAAAAGTCTTGCGCA |
| *Plaur* | CTACCTGTGTCCCAGCCTCCCA | CCCGAAGCACGGTAGTCCTGC |
| *Rbl2* | TGCAGCCAGCTCGGAGGAAG | CATTTCCCTCCAGCGTGTAGCTCT |
| *Serpine1* | TGGCGTCTTCCTCCACAGCCTT | TGTCTCTGTCGGGTTGTGCCG |
| *Skp2* | TGGTACCGCCTCTCGCTCGAT | GCGCACAGTCACGTCTGGGT |
