# Supplementary material for: Xenobiotic-Induced Hepatocyte Proliferation Associated with Constitutive Active/Androstane Receptor (CAR) or Peroxisome Proliferator-Activated Receptor α (PPARα) Is Enhanced by Pregnane X Receptor (PXR) Activation in Mice
Source: PLoS One. 2013 Apr 23;8(4):e61802. doi: 10.1371/journal.pone.0061802 (PMC3634023; doi:10.1371/journal.pone.0061802)
Supplement: Table S2 — Changes in the gene expression levels after PCN and/or TCPOBOP treatment in mouse livers. (DOC) [file pone.0061802.s003.doc]

**Table S2.** **Changes in the gene expression levels after PCN and/or TCPOBOP treatment in mouse livers.**

| Gene symbol | Description | Fold change (vs Control) | | |
| --- | --- | --- | --- | --- |
|  |  | PCN | TC | TC+PCN |
| *Abl1* | C-abl oncogene 1, receptor tyrosine kinase | 0.77 | 0.67 | 0.63 |
| *Ak1* | Adenylate kinase 1 | 0.77 | 0.54 | 0.7 |
| *Atm* | Ataxia telangiectasia mutated homolog (human) | 0.45 | 0.45 | 0.48 |
| *Brca1* | Breast cancer 1 | 1.27 | 1.02 | 2.64 |
| *Brca2* | Breast cancer 2 | 0.97 | 1.3 | 2.33 |
| *Camk2b* | Calcium/calmodulin-dependent protein kinase II, beta | 0.46 | 0.34 | 0.26 |
| *Ccna2* | Cyclin A2 | 0.48 | 1.92 | 3.28 |
| *Ccnb1* | Cyclin B1 | 0.41 | 2.61 | 5.32 |
| *Ccnb2* | Cyclin B2 | 0.49 | 1.96 | 4.67 |
| *Ccnc* | Cyclin C | 0.38 | 0.73 | 0.52 |
| *Ccne1* | Cyclin E1 | 0.59 | 0.62 | 1.26 |
| *Ccnf* | Cyclin F | 0.83 | 0.74 | 0.57 |
| *Cdk5rap1* | CDK5 regulatory subunit associated protein 1 | 0.62 | 0.63 | 0.48 |
| *Cdkn1a* | Cyclin-dependent kinase inhibitor 1A (P21) | 0.25 | 0.56 | 0.9 |
| *Cdkn1b* | Cyclin-dependent kinase inhibitor 1B (P27) | 0.90 | 0.66 | 0.52 |
| *Chek1* | Checkpoint kinase 1 homolog (S. pombe) | 1.27 | 1.02 | 1.95 |
| *Cks1b* | CDC28 protein kinase 1b | 1.19 | 1.96 | 1.77 |
| *Ddit3* | DNA-damage inducible transcript 3 | 1.01 | 0.68 | 0.61 |
| *Dnajc2* | DnaJ (Hsp40) homolog, subfamily C, member 2 | 0.54 | 0.60 | 0.75 |
| *Dst* | Dystonin | 0.78 | 0.63 | 0.78 |
| *E2f1* | E2F transcription factor 1 | 1.15 | 1.44 | 2.18 |
| *Gadd45a* | Growth arrest and DNA-damage-inducible 45 alpha | 1.54 | 3.35 | 5.95 |
| *Gpr132* | G protein-coupled receptor 132 | 0.78 | 0.86 | 0.58 |
| *Mad2l1* | MAD2 mitotic arrest deficient-like 1 (yeast) | 0.7 | 1.07 | 1.53 |
| *Mcm2* | Minichromosome maintenance deficient 2 mitotin (S. cerevisiae) | 1.27 | 2.35 | 4.23 |
| *Mcm3* | Minichromosome maintenance deficient 3 (S. cerevisiae) | 1.06 | 1.14 | 2.33 |
| *Mcm4* | Minichromosome maintenance deficient 4 homolog (S. cerevisiae) | 0.76 | 0.96 | 1.71 |
| *Mki67* | Antigen identified by monoclonal antibody Ki 67 | 0.57 | 2.02 | 3.28 |
| *Mtbp* | Mdm2, transformed 3T3 cell double minute p53 binding protein | 1.09 | 1.01 | 1.87 |
| *Nek2* | NIMA (never in mitosis gene a)-related expressed kinase 2 | 1.22 | 1.96 | 4.93 |
| *Nfatc1* | Nuclear factor of activated T-cells, cytoplasmic, calcineurin-dependent 1 | 1.02 | 0.71 | 0.65 |
| *Pcna* | Proliferating cell nuclear antigen | 0.89 | 1.36 | 1.62 |
| *Pkd1* | Polycystic kidney disease 1 homolog | 0.89 | 0.68 | 0.57 |
| *Ppm1d* | Protein phosphatase 1D magnesium-dependent, delta isoform | 0.97 | 0.66 | 0.58 |
| *Ppp2r3a* | Protein phosphatase 2 (formerly 2A), regulatory subunit B'', alpha | 0.75 | 0.6 | 0.49 |
| *Ppp3ca* | Protein phosphatase 3, catalytic subunit, alpha isoform | 0.59 | 0.57 | 0.46 |
| *Rad17* | RAD17 homolog (S. pombe) | 0.49 | 0.59 | 0.64 |
| *Rad51* | RAD51 homolog (S. cerevisiae) | 0.81 | 2.23 | 6.83 |
| *Ran* | RAN, member RAS oncogene family | 1.21 | 1.63 | 1.53 |
| *Rbl1* | Retinoblastoma-like 1 (p107) | 1.09 | 1.08 | 1.79 |
| *Rbl2* | Retinoblastoma-like 2 (p130) | 0.9 | 0.83 | 0.65 |
| *Skp2* | S-phase kinase-associated protein 2 (p45) | 1.06 | 0.87 | 2.04 |
| *Terf1* | Telomeric repeat binding factor 1 | 0.66 | 0.60 | 0.65 |

Total RNA used in Fig. 1D was pooled and subject to PCR-array analyses. Genes, whose expression levels were changed more than 1.5-fold by treatment with TCPOBOP or co-treatment with TCPOBOP and PCN, are shown. TC, TCPOBOP.
